# Comparative Efficacy of Various Stents for Palliation in Patients with Malignant Extrahepatic Biliary Obstruction: A Systematic Review and Network Meta-Analysis

**DOI:** 10.3390/jpm11020086

**Published:** 2021-01-30

**Authors:** Chan Hyuk Park, Se Woo Park, Jang Han Jung, Eun Suk Jung, Jung Hee Kim, Da Hae Park

**Affiliations:** 1 Department of Internal Medicine, Hanyang University Guri Hospital, Hanyang University College of Medicine, Guri 11923, Korea; yesable7@gmail.com; 2 Division of Gastroenterology, Department of Internal Medicine, Hallym University Dongtan Sacred Heart Hospital, Hallym University College of Medicine, Hwaseong 18450, Korea; con2000@hallym.or.kr (J.H.J.); esjung@hallym.or.kr (E.S.J.); jungheekim@hallym.or.kr (J.H.K.); dahaepark82@gmail.com (D.H.P.)

**Keywords:** malignant biliary obstruction, stent, plastic stent, metal stent, recurrent biliary obstruction

## Abstract

Although many studies have investigated the efficacy of stent placement for patients with malignant extrahepatic biliary obstruction, the clinical outcomes and adverse events of biliary stenting have not been comprehensively evaluated. We searched all relevant randomized-controlled trials that evaluated the comparative efficacy of biliary stents, including the plastic stents, uncovered self-expandable metal stents (SEMSs), and covered SEMSs in patients with malignant extrahepatic biliary obstructions. Twenty-one studies with 2326 patients were included. Both uncovered and covered SEMSs had a lower risk of recurrent biliary obstruction (RBO) compared to plastic stents (risk ratio (RR) (95% confidence interval [CI]): uncovered vs. plastic, 0.46 (0.35–0.62); covered vs. plastic, 0.46 (0.34–0.62)). A comparison of the groups using SEMSs revealed that tumor ingrowth was common in the uncovered SEMS group, while stent migration, tumor overgrowth, and occlusion by sludge were common in the covered SEMS group; however, the overall risk of RBO did not differ between these groups (RR (95% CI): uncovered vs. covered: 1.02 (0.80–1.30)). Although the main causes of RBO vary across stents, RBO risk was similar between uncovered and covered SEMS groups. Both SEMSs have superior efficacy in terms of RBO compared to plastic stents.

## 1. Introduction

Appropriate biliary drainage is important for relieving symptoms and extending life expectancy in patients with unresectable malignant biliary obstruction (MBO) [1]. Biliary drainage techniques involving endoscopic retrograde cholangiopancreatography (ERCP) or percutaneous transhepatic biliary drainage were established approximately 40 years ago [2,3]. In the 1980s, when endoscopic drainage was first introduced, plastic stents were typically used for the procedure [4]. Biliary drainage using plastic stents was as effective as surgical drainage [2,4]. In the 1990s, self-expandable metal stents (SEMSs) were introduced, and their efficacies were compared with those of plastic stents in many studies [5]. Endoscopic drainage using SEMSs prolongs patient survival, lowers adverse events, including recurrent biliary obstruction (RBO) [6], and requires fewer re-interventions compared with drainage using plastic stents [5,7]. Although SEMSs are more expensive than plastic stents, a recent randomized-controlled trial (RCT) showed that the total costs up to one year after stenting did not significantly differ between plastic stent and SEMS groups because of the longer functional time of SEMSs [8].

Regarding subtypes of SEMSs, several meta-analyses compared the efficacies of uncovered and covered SEMSs during endoscopic drainage for patients with extrahepatic MBO [9,10]. According to these studies, using covered SEMSs entailed a lower risk of tumor ingrowth, but a higher risk of tumor overgrowth, stent migration, and occlusion by sludge or food crap, compared to the use of uncovered SEMSs. RBO, overall adverse events, and the overall survival period did not differ between both SEMSs [9,10]. 

Although several meta-analyses evaluated differences among the efficacies of stent types, it is still difficult to comprehensively understand this topic as previous meta-analyses focused on pairwise comparisons between two stent types (e.g., plastic vs. metal stents, and uncovered vs. covered SEMSs) that would provide only fragmented comparative efficacies [5,7,9,10]. Additionally, without knowing absolute efficacies, it is difficult to know how many patients can avoid stent-related adverse events depending on stent types. Therefore, we performed a network meta-analysis of RCTs comparing efficacies of the different types of stents in patients with extrahepatic MBO. We also investigated the absolute risk of clinical outcomes and stent-related adverse events based on all relevant RCTs. 

## 2. Materials and Methods

### 2.1. Study Design

We performed a systematic review and network meta-analysis in adherence to the Preferred Reporting Items for Systematic Reviews and Meta-Analyses statement [11] and the International Society for Pharmacoeconomics and Outcomes Research Task Force on Indirect Treatment Comparisons Good Research Practices report [12]. 

### 2.2. Search Strategy

All relevant studies published between January 1990 and January 2020 that evaluated the efficacy of stents for palliation in patients with extrahepatic MBO were retrieved from the MEDLINE, EMBASE, and Cochrane Library databases. The following search string was used: ((bile duct) or (biliary) or (cholangiocarcinoma) or (cholangiocarcinomas) or (pancreatic) or (pancreas)) and ((obstruction) or (obstructive) or (stricture) or (strictures) or (jaundice) or (cholestasis)) and ((nonresectable) or (unresectable) or (palliation) or (palliative) or (palliating) or (inoperable)) and ((stent) or (stents) or (stenting) or (endoprostheses)) and (random*). Appendix A shows the detailed search strategies for each database. Additionally, we examined the references of the screened articles to identify additional relevant studies. Our search was last updated at 15 January 2020.

### 2.3. Inclusion/Exclusion Criteria

The inclusion criteria were as follows: (a) population: patients with unresectable extrahepatic MBO, (b) intervention: ERCP with palliative biliary decompression using stents including a plastic, uncovered, or covered stent, (c) comparator: ERCP with palliative biliary decompression with another type of stent, and (d) outcome: technical and clinical success, RBO, and adverse events. Non-human studies, non-original studies, non-RCTs, abstract-only publications, and studies published in languages other than English were excluded.

### 2.4. Study Selection

In the first step of the study selection, duplicated articles, which were retrieved through multiple search engines, were excluded. Thereafter, we examined the titles and abstracts of the articles to exclude irrelevant studies. The full text of the remaining articles was then assessed for eligibility. Two investigators (C.H.P. and S.W.P.) independently evaluated the studies for eligibility and resolved any disagreements through discussion. If an agreement could not be reached, a third investigator (J.H.J.) determined study eligibility. The Cochrane Risk of Bias assessment tool was used for assessing the risk of bias in the included RCTs.

### 2.5. Data Extraction and Study Endpoint

Using a data extraction form developed in advance, two reviewers (C.H.P. and S.W.P.) independently extracted the following information: first author name, year of publication, study design, country, study period, publication language, type of biliary stent (plastic stent, uncovered SEMS, or covered SEMS), and clinical outcomes, including clinical success, RBO, and adverse events. 

The primary endpoint in this meta-analysis was the comparative efficacy of biliary stents in terms of RBO. Secondary endpoints were causes of RBO, including occlusion by sludge, tumor ingrowth and overgrowth, and stent migration, and stent-related inflammation, including cholangitis, cholecystitis, and pancreatitis.

### 2.6. Statistical Analysis

A direct pairwise meta-analysis was conducted to calculate the risk ratios (RRs) of categorical variables, including those of RBO and adverse events using a random-effects model. For technical and clinical successes, we calculated crude proportions rather than RRs because the technical or clinical success rates were 100% in many studies, and those studies could not be included for calculating pooled RRs. Statistical heterogeneity was assessed using two methods: Cochrane’s Q test, in which *p*-values of <0.1 were considered statistically significant for heterogeneity, and *I^2^* statistics, wherein values of >50% suggested significant heterogeneity [13]. We assessed publication bias quantitatively using the Begg and Mazumdar adjusted-rank correlation test (publication bias was considered present if *p* was <0.1) [14]. We also assessed bias qualitatively by inspecting the funnel plots of logarithmic RRs versus their standard errors [15]. When the number of included studies for each pairwise comparison was less than 10, the test for publication bias was not conducted [16]. A direct pairwise meta-analysis was performed using the Review Manager statistical software (version 5.3.5; Cochrane Collaboration, Copenhagen, Denmark) and Comprehensive Meta Analysis (version 2.2.064; Biostat Inc., Englewood, NJ, USA) software.

A frequentist network meta-analysis was performed to calculate direct and indirect estimates and combine mixed estimates [17]. Moreover, each stent type was ranked according to P-scores, which were based solely on the point estimates and standard errors of network estimates [18]. The P-score of each eradication regimen can be interpreted as the mean extent of certainty that a certain regimen was better than another [18]. The network meta-analysis was performed using the R statistical software (version 3.6.2; R Foundation for Statistical Computing, Vienna, Austria) with the *netmeta* package (version 0.9–1; Rücker et al.). The *netmeta* package is based on the graph theory methodology used to model the relative treatment effects of multiple treatments under a frequentist framework [19].

## 3. Results

### 3.1. Study Selection and Characteristics

**Figure 1:**
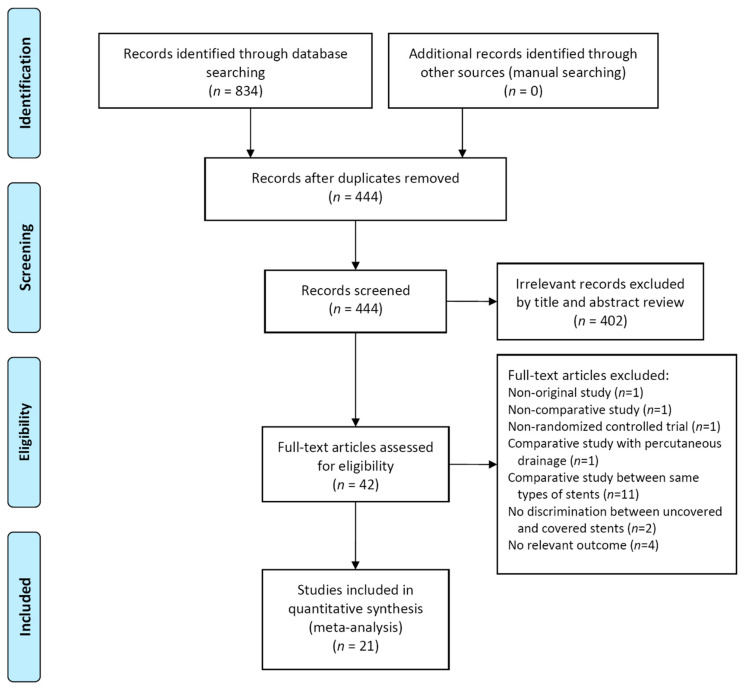
Flow diagram of the studies included in the meta-analysis.

Twenty-one studies including 2326 patients were included in our meta-analysis (Figure 1) [8,20,21,22,23,24,25,26,27,28,29,30,31,32,33,34,35,36,37,38,39]. 

The baseline patient and lesion characteristics are summarized in Table 1. Studies were published between 1992 and 2020 with an enrollment period that ranged from 1990 to 2018. Six studies evaluated compared the efficacy of plastic stents and that of uncovered SEMSs [20,21,22,23,25,37], while three studies compared the efficacy of plastic stents and that of covered SEMSs [26,30,33]. The other 11 studies compared the efficacy of uncovered SEMSs and that of covered SEMSs [24,27,28,29,31,32,34,35,36,38,39]. The remaining study was a three-arm trial, which compared the efficacy of plastic stents, that of uncovered SEMS, and that of covered SEMS [8]. 

The evidence network is shown in Figure S1. 

Lines represent the comparison between stent types. The thickness of these lines and the numbers shown in large represent the number of studies included in each comparison. The numbers in rectangles indicate the number of patients included in each comparison. Because one study was a three-arm trial, the sum of comparisons and that of patients exceeded the number of included studies and that of included patients, respectively. SEMS, self-expandable metal stent.

The risk of bias assessments for individual studies is shown in Figure S2. 

Among the 21 included studies, 3 (14.3%) had an unclear risk of bias regarding random sequence generation. An unclear risk of bias in terms of allocation concealment was identified in 8 studies (38.1%). All studies were assessed as having a low risk of performance and detection bias because stent-related outcomes, including RBO, are less likely to be affected by the blinding of participants and investigators. Attrition bias was not identified. One study (4.8%) was assessed as having a high risk of reporting bias because it did not report the proportion of RBO, which was a primary endpoint of our meta-analysis. 

### 3.2. Technical and Clinical Success According to the Stent Type

Technical and clinical successes were excellent regardless of stent type. The proportion of technical success was 99.4% (95% CI, 98.6–99.7%) in the plastic stent group, 100.0% (95% CI, 98.5–100.0%) in the uncovered SEMS group, and 99.2% (95% CI, 98.6–99.7%) in the covered SEMS group. Additionally, the proportion of clinical success was 90.1% (95% CI, 84.9–93.6%) in the plastic stent group, 94.6% (95% CI, 91.2–96.7%) in the uncovered SEMS group, and 92.3% (95% CI, 88.4–94.9%) in the covered SEMS group. There was no statistical difference in technical and clinical successes among stent types.

### 3.3. Direct Meta-Analysis for Recurrent Biliary Obstruction and Stent-Related Inflammation

**Figure 2:**
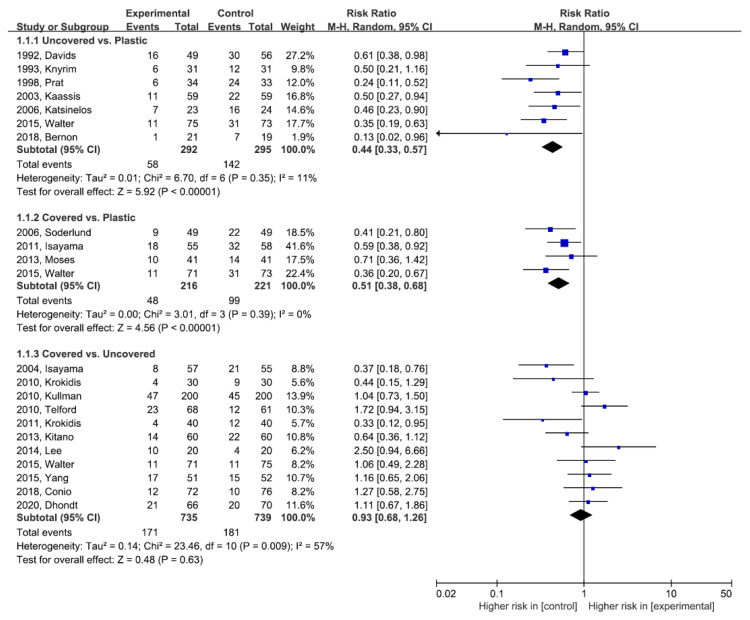
Direct meta-analysis for recurrent biliary obstruction. M-H, Mantel–Haenszel; CI, confidence interval.

Figure 2 demonstrates the comparative efficacy in terms of RBO between any two stent types. Both the uncovered and covered SEMS groups showed a lower risk of RBO compared to the plastic stent group without statistically significant heterogeneity (uncovered SEMS vs. plastic stent: RR (95% CI) = 0.44 (0.33–0.57), df = 6, *p* = 0.35, *I^2^* = 11%; and covered SEMS vs. plastic stent: RR (95% CI) = 0.51 (0.38–0.68), df = 3, *p* = 0.39, *I^2^* = 0%). 

Between the uncovered and covered SEMS groups, there was no significant difference in terms of RBO, but there was statistical heterogeneity (covered SEMS vs. uncovered SEMS: RR (95% CI) = 0.93 (0.68–1.26), df = 10, *p* = 0.009, *I^2^* = 57%). Publication bias was assessed for RBO between the uncovered and covered SEMS groups. We did not detect any corresponding publication bias using the Begg and Mazumdar-adjusted rank correlation tests (*p* = 0.938). Asymmetry was not observed upon the visual inspection of the funnel plot (Figure S3). 

The white diamond represents the pooled logarithmic RR, with a 95% confidence interval, among observed studies.

RBO, recurrent biliary obstruction; SEMS, self-expandable metal stent; M-H, Mantel–Haenszel; RR, risk ratio

Figure S4 shows forest plots for causes of RBO, including stent occlusion by sludge, tumor ingrowth, tumor overgrowth, and stent migration. Stent occlusion by sludge was common in the plastic stent group, tumor ingrowth was common in the uncovered SEMS group, and tumor overgrowth and stent migration tended to be common in the covered SEMS group. M-H, Mantel–Haenszel; CI, confidence interval.

In terms of stent-related inflammation, there was no statistical difference in most comparisons (Figure S5). Although cholangitis was less common in the covered SEMS group than in the plastic stent group, only one study was included in that comparison. M-H, Mantel–Haenszel; CI, confidence interval.

### 3.4. Network Meta-Analysis for Recurrent Biliary Obstruction and Stent-Related Inflammation

**Figure 3:**
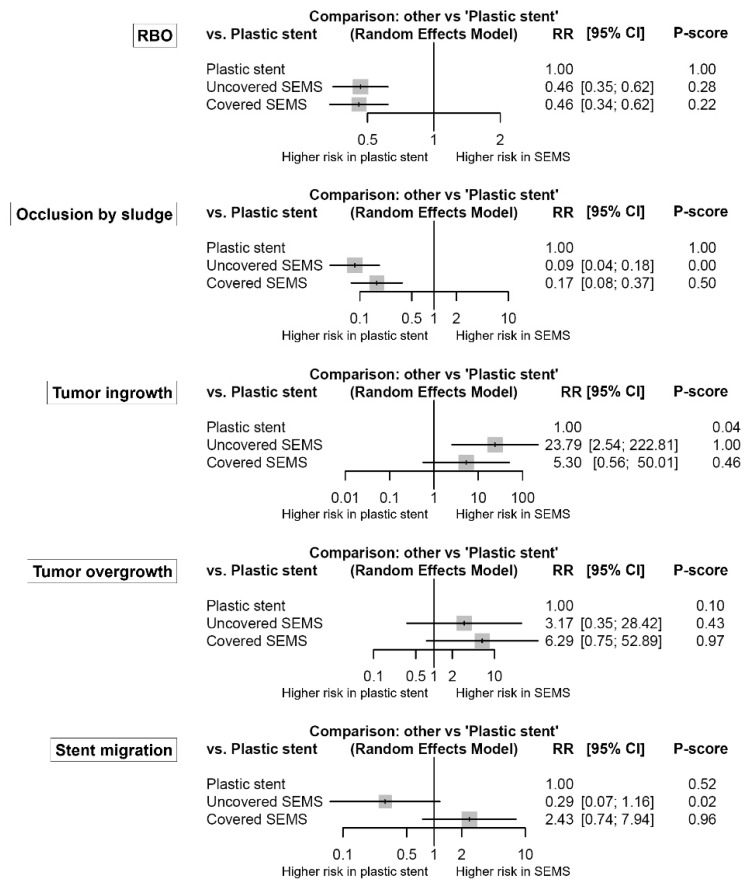
Comparative efficacy for recurrent biliary obstruction and its causes according to the type of stents from the network meta-analysis. The P-score indicates the mean extent of certainty that one type of stent is better than another. RBO, recurrent biliary obstruction; SEMS, self-expandable metal stent; RR, risk ratio; CI, confidence interval.

In Figure 3, forest plots show the network estimates of RBO and its causes in the uncovered and covered SEMS groups compared with the plastic stent group. Both uncovered and covered SEMS groups showed a lower risk of RBO compared to the plastic stent group (RR (95% CI): uncovered SEMS vs. plastic stent, 0.46 (0.35–0.62); and covered SEMS vs. plastic stent, 0.46 (0.34–0.62)). There was no significant difference between the uncovered and covered SEMS groups (uncovered vs. covered: RR (95% CI) = 1.02 (0.80–1.30)). Plastic stents had the highest P-score (>99%), followed by uncovered SEMS (28%) and covered SEMS (22%). 

The detailed efficacy profiles of stents are summarized in Table S1. There was no network inconsistency in the comparison.

Both uncovered and covered SEMS groups also had a lower risk of stent occlusion by sludge compared to the plastic stent group (RR (95% CI): uncovered SEMS vs. plastic stent, 0.09 (0.04–0.18); and covered SEMS vs. plastic stent, 0.17 (0.08–0.37)). Additionally, the risk of stent occlusion by sludge in the uncovered SEMS group was lower than that in the covered SEMS group (uncovered vs. covered: RR (95% CI) = 0.51 (0.31–0.83)). However, the uncovered SEMS group had a higher risk of tumor ingrowth compared to the covered SEMS and plastic stent groups (RR (95% CI): uncovered SEMS vs. covered SEMS, 4.49 (2.21–9.09); and uncovered SEMS vs. plastic stent, 23.79 (2.54–222.81)). In terms of tumor overgrowth, both uncovered and covered SEMS groups tended to be higher risk compared to the plastic stent group. However, the covered SEMS group had a significantly higher risk of tumor overgrowth compared to the uncovered SEMS group (covered vs. uncovered: RR (95% CI) = 1.98 (1.18–3.34)). Stent migration was common in the covered SEMS group than in the uncovered SEMS group (covered vs. uncovered: RR (95% CI) = 8.38 (2.83–24.84)). There was no network inconsistency in any comparison.

**Figure 4:**
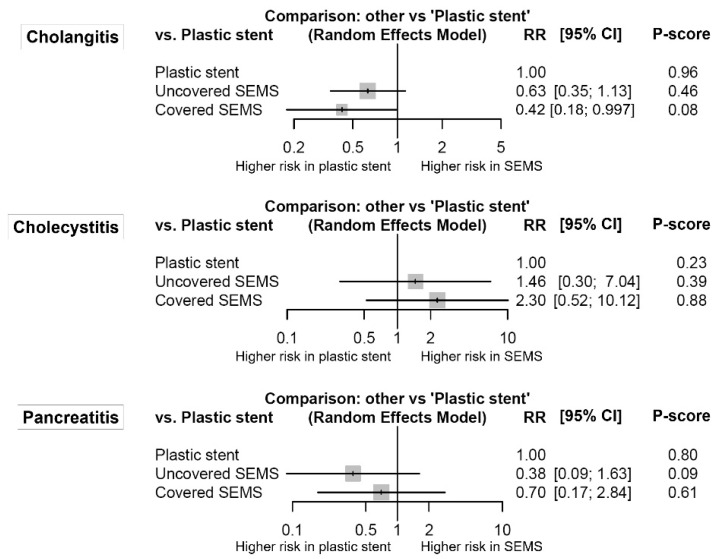
Comparative efficacy for stent-related inflammation according to the type of stents from the network meta-analysis. The P-score indicates the mean extent of certainty that one type of stent is better than another. SEMS, self-expandable metal stent; RR, risk ratio; CI, confidence interval.

Figure 4 shows the forest plots for stent-related inflammation. Although cholangitis was marginally more common in the plastic stent group than in the covered SEMS group (RR (95% CI) = 2.37 (1.003–5.60)), there was no significant difference or network inconsistency in any other comparison.

### 3.5. Absolute Risk of Recurrent Biliary Obstruction and Adverse Events

To understand the absolute risk of RBO and adverse events, the incidence of each outcome was summarized in Table S2. The proportion of RBO was 47.4% (95% CI, 42.8–52.1%) in the plastic stent group, 23.8% (95% CI, 21.3–26.7%) in the uncovered SEMS group, and 23.6% (95% CI, 20.9–26.6%) in the covered SEMS group. RBO was mainly caused by sludge in the plastic stent group (incidence, 44.8% (95% CI, 37.3–52.5%)) and tumor ingrowth in the uncovered SEMS group (incidence, 17.3% (95% CI, 14.5–20.4%)). In the covered SEMS group, stent occlusion by sludge, tumor overgrowth, and stent migration was found in 7.5%, 7.1%, and 5.0% of patients, respectively. The proportion of cholangitis was 36.3% (95% CI, 29.4–43.7%) in the plastic stent group, 12.1% (95% CI, 9.5–15.4%) in the uncovered SEMS group, and 5.2% (95% CI, 3.4–7.9%) in the covered SEMS group.

## 4. Discussion

This network meta-analysis study is the first to comprehensively compare three types of stents for the endoscopic drainage of extrahepatic MBO. Compared to the traditional pairwise meta-analytic approach, our study has the innovative advantage of filling the literature gap between RCTs, offering the application of appropriate stents to the clinical field through novel comparisons. We showed that all three types of stents have extremely high technical success rates of more than 99%. Additionally, all stent types showed high clinical success rates of more than 90%. However, the clinical success rate of the plastic stent tended to be 4.5% lower than that of the uncovered SEMS (90.1% vs. 94.6%). This might be derived from the vulnerability of the plastic stent and sludge occlusion, followed by biliary obstruction, subsequent cholangitis, and the need for stent revision [40,41]. 

Regarding primary outcomes related to RBO, both uncovered and covered SEMSs showed a lower risk compared to plastic stents. The diameter of the stent may play an essential role in maintaining stent patency; thus, a 10-Fr plastic stent would be better than 7- or 8-Fr stents [42]. However, duodenoscopes have a limitation of delivery for plastic stents greater than about 11.5-Fr stents through the working channel. In contrast, biliary SEMSs with larger internal diameters (up to 10 mm) can be placed with a 7- or 8-Fr delivery system, which is enough to pass through the working channel [43]. 

RBO did not differ between uncovered and covered SEMSs. However, it was affected by different mechanisms. Covered SEMSs were more likely to result in RBO from sludge occlusion, tumor overgrowth, and stent migration, whereas uncovered SEMSs were mainly influenced by tumor ingrowth. The coating membrane of the covered SEMS was invented to prevent tumor ingrowth; however, it offers a medium for the attachment and colonization of a bacterial biofilm [9], which lead to stent sludge occlusion. Simultaneously, it can disturb the embedding and anchoring of the stent in situ [44]. This can consequently lead to an 8.4-fold higher chance of stent migration compared with uncovered SEMSs. However, the absolute risk of stent migration was relatively low even in patients who underwent covered SEMS placement (5.0%) compared with that of tumor ingrowth caused by uncovered SEMSs (17.3%). 

Although SEMSs have a relatively good stent patency, their revision for RBO is challenging compared to plastic stents because of difficulties in stent removal or replacement, especially when an uncovered SEMS was placed. Thus, plastic stents might be appropriate for patients with a life expectancy of less than six months, whereas SEMS can be more favorable for patients with a life expectancy of six months or more [45]. 

Among stent-related inflammations, including cholangitis, cholecystitis, and pancreatitis, cholangitis may be the most concerning because it is the most common. The risk of cholangitis in the plastic stent group was higher than that in the covered SEMS group. Because biliary drainage through plastic stents may require more re-interventions than drainage through SEMSs, the risk of cholangitis can be increased when plastic stents are placed. Regarding cholecystitis, several studies reported that covered SEMSs might have a higher cholecystitis risk after biliary stent placement than after uncovered SEMS placement [46,47]. Additionally, our meta-analysis showed a tendency of increased cholecystitis risk in the covered SEMS group compared to the plastic stent group; however, there was no significant difference between groups. It was difficult to identify the small differences regarding cholecystitis risk among stents because incidences of stent-related cholecystitis were very low (0.5% for plastic stents, 1.6% for uncovered SEMSs, and 3.1% for covered SEMSs). Additionally, it has been suggested that tumor invasions to the feeding artery of the gallbladder or the orifice of the cystic duct, rather than the stent itself, might contribute to the development of cholecystitis [48]. Meanwhile, there is also a concern regarding acute pancreatitis surrounding SEMSs because the opening of the pancreatic duct could, theoretically, be occluded by the axial force of the stent [49,50]. However, our analysis showed no statistical difference in the risk of pancreatitis among stents.

Although this study was the first network meta-analysis to compare the efficacies of various stents in the palliative drainage for extrahepatic MBO, it has several limitations. First, significant heterogeneity was identified in the comparisons of uncovered and covered SEMSs regarding RBO and tumor ingrowth; however, there was no significant heterogeneity in any other comparisons. Second, the specific models of the compared stents varied across the included studies. Although the inclusion of various stent models may help generalize our study findings, it may be a potential confounding variable. A multicenter-cohort study reported that covered WallFlex stents were superior to covered Wallstents in terms of the cumulative incidence of RBO [51]. A Korean multicenter study showed different migration risk between covered SEMS with flaps and conventional covered SEMS in patients with benign biliary strictures [52]. Third, biliary obstructions in the included patients were caused by various underlying malignant diseases, including pancreatic cancer, bile duct cancer, gallbladder cancer, and ampullary cancer. Because clinical outcomes and adverse events in each included study were reported without the consideration of underlying diseases, we could not perform a subgroup analysis according to the types of malignant diseases. 

Despite these limitations, the present network meta-analysis provides a better understanding of clinical outcomes and adverse events according to the type of stents in patients with extrahepatic MBO. In conclusion, we recommend that the use of an SEM should be considered as the first option to treat extrahepatic MBOs because SEMs have longer stent patency, lower sludge occlusion rate, and lower risk of stent-related cholangitis, compared with plastic stents. Uncovered SEMSs had a high risk of tumor ingrowth, whereas covered SEMSs had a high risk of stent migration, tumor overgrowth, and sludge occlusion. However, the overall risk of RBO was similar between the uncovered and covered SEMSs.

## Figures and Tables

**Table 1:** Baseline patient and lesion characteristics in the included studies.

First Author	Publication Year	Country	Study Period	Number of Patients	Age, Year, Mean ± SD	Male, n (%)	Performance Status	Cause of Obstruction	Baseline total Bilirubin, mg/dL, Mean ± SD	Used Stent
Davids	1992	Netherlands	1990–1992	Plastic: 56 Uncovered: 49	Plastic: 76.5 (range, 53–96) Uncovered: 76 (range, 46–94)	Plastic: 41.1 Uncovered: 49.0	N/A	Plastic: pancreatic cancer (89.3%), ampullary cancer (10.7%) Uncovered: pancreatic cancer (87.8%), ampullary cancer (12.2%)	Plastic: median 10.2 (range, 1.1–34.2) Uncovered: median 11.3 (range, 0.7–41.6)	Plastic: 10-Fr endoprostheses (PBN Medicals, Stenloese, Denmark) Uncovered: 30-Fr metal stent (Wallstent, Schneider, Switzerland)
Knyrim	1993	Germany	1990–1992	Plastic: 31 Uncovered: 31	Plastic: 70.2 ± 1.8 Uncovered: 70.8 ± 2.0	N/A	ECOG performance status Plastic: median 3 Uncovered: median 3	Plastic: pancreatic cancer (71.0%), bile duct cancer (3.2%), ampullary cancer (3.2%), others (22.6%) Uncovered: pancreatic cancer (67.7%), bile duct cancer (3.2%), ampullary cancer (6.5%), others (22.6%)	Plastic: median 10 Uncovered: median 12	Plastic: 11.5-Fr polyethylene plastic stent (Huibregtse biliary stent, Wilson-Cook, Winston-Salem, NC, USA) Uncovered: Wallstent (Schneider AG, Bülach, Switzerland) or Strecker-Stent (Boston Scientific, Watertown, MA, USA)
Prat	1998	France	1993–1995	Plastic: 33 Uncovered: 34	Plastic: mean 73.3 (range, 39–92) Uncovered: mean 71.9 (range, 39–95)	Plastic: 51.5 Uncovered: 44.1	N/A	Plastic: pancreatic cancer (66.7%), bile duct cancer (21.2%), ampullary cancer (3.0%), metastatic cancer (9.1%) Uncovered: pancreatic cancer (73.5%), bile duct cancer (14.7%), ampullary cancer (0.0%), metastatic cancer (11.8%)	Plastic: mean 13.2 Uncovered: mean 14.7	Plastic: 11.5-Fr polyethylene stent (Wilson Cook, Winston-Salem, NC, USA) Uncovered: 30-Fr self-expanding metallic stent (Wallsent, Schneider-Howmedica, Lyons, France)
Kaassis	2003	France	1997–1999	Plastic: 59 Uncovered: 59	Plastic: median 75.9 (IQR, 66.9–84.3) Uncovered: median 78.5 (IQR, 70.0–87.9)	Plastic: 54.2 Uncovered: 35.6	N/A	Plastic: pancreatic cancer (72.9%), bile duct cancer (13.6%), metastatic cancer (11.9%), others (1.7%) Uncovered: pancreatic cancer (76.3%), bile duct cancer (16.9%), metastatic cancer (5.1%), others (1.7%)	Plastic: median 16.2 (IQR, 7.7–20.2) Uncovered: median 13.7 (IQR, 6.7–18.0)	Plastic: 10-Fr Tannenbaum-type stent (Soehendra ST-2, Wilson Cook, Clarenton, France) Uncovered: 10-mm metal stent (Wallstent, Boston Scientific Corp., St. Quentin en Yvelines, France)
Isayama	2004	Japan	1998–2001	Uncovered: 55 Covered: 57	Uncovered: mean 70.4 (range, 40–89) Covered: mean 70.5 (range, 48–88)	Uncovered: 56.4 Covered: 61.4	N/A	Uncovered: pancreatic cancer (58.2%), bile duct cancer (9.1%), gallbladder cancer (10.9%), ampullary cancer (1.8%), metastatic cancer (20.0%) Covered: pancreatic cancer (59.6%), bile duct cancer (10.5%), gallbladder cancer (5.3%), ampullary cancer (3.5%), metastatic cancer (21.1%)	Uncovered: 8.5 ± 6.3 Covered: 10.5 ± 7.2	Uncovered: Uncovered Ultraflex Diamond Stent (Microvasive; Boston Scientific Corporation, Natik, Massachusetts, USA) Covered: Self-expandable Ultraflex Diamond Stent (Microvasive, Boston Scientific Corporation, Natik, Massachusetts, USA)
Katsinelos	2006	Greece	2000–2005	Plastic: 24 Uncovered: 23	Plastic: median 72 (range, 56–82) Uncovered: median 74 (range, 57–86)	Plastic: 41.7 Uncovered: 60.9	ASA PS classification Plastic (II/III): 45.8%/54.2% Uncovered (II/III): 39.1%/60.9%	Plastic: pancreatic cancer (54.2%), bile duct cancer (16.7%), ampullary cancer (12.5%), metastatic cancer (16.7%) Uncovered: pancreatic cancer (52.2%), bile duct cancer (17.4%), ampullary cancer (8.7%), metastatic cancer (21.7%)	Plastic: median 10.6 (range, 4.8–19.3) Uncovered: median 14.1 (range, 5.4–35.8)	Plastic: Teflon Tannenbaum stents (Sohendra ST-2, Wilson Cook, Winston-Salem, NC, USA) Uncovered: uncovered self-expanding metal stent (M.I. Tech, G.C. Medical Co. Ltd., Pyungtaek, Korea)
Soderlund	2006	Sweden	2002–2004	Plastic: 51 Covered: 49	Plastic: median 78 (range, 49–93) Covered: median 77 (range, 48–92)	Plastic: 54.9 Covered: 44.9	ECOG performance status Plastic (0/1/2/3/4): 15.7%/37.3%/31.4%/11.8%/3.9% Covered (0/1/2/3/4): 16.3%/40.8%/28.6%/10.2%/4.1%	Plastic: pancreatic cancer (74.5%), bile duct cancer (7.8%), ampullary cancer (2.0%), metastatic cancer (9.8%), others (5.9%) Covered: pancreatic cancer (81.6%), bile duct cancer (10.2%), ampullary cancer (2.0%), metastatic cancer (4.1%), others (2.0%)	Plastic: median 13.9 (range, 0.5–36.8) Covered: median 11.6 (range, 1.1–40.1)	Plastic: 10-Fr polyethylene endoprothesis (Boston Scientific Nordic AB, Helsingborg, Sweden) Covered: Wallstent (Boston Scientific Nordic AB, Helsingborg, Sweden)
Krokidis	2010	Italy and Greece	2005–2007	Uncovered: 30 Covered: 30	Uncovered: median 63.7 (range, 46–73) Covered: median 66.5 (range, 52–78)	Uncovered: 53.3 Covered: 66.7	N/A	Uncovered: bile duct cancer (100.0%) Covered: bile duct cancer (100.0%)	Uncovered: 7.2 Covered: 10.3	Uncovered: 10-mm Uncoverd Wallstent Covered: 8- to 10-mm Viabil Biliary Stent
Kullman	2010	Sweden	2006–2008	Uncovered: 200 Covered: 200	Uncovered: median 76 (range, 51–95) Covered: median 79 (range, 39–100)	Uncovered: 45.5 Covered: 44.0	ECOG performance status Uncovered (0/1/2/3/4): 21.0%/24.0%/37.0%/15.0%/3.0% Covered (0/1/2/3/4): 23.5%/23.5%/38.5%/13.5%/1.0%	Uncovered: pancreatic cancer (77.5%), bile duct cancer (5.0%), gallbladder cancer (1.5%), ampullary cancer (4.5%), metastatic cancer (9.0%), others (2.5%) Covered: pancreatic cancer (76.0%), bile duct cancer (6.0%), gallbladder cancer (4.0%), ampullary cancer (4.0%), metastatic cancer (8.0%), others (2.0%)	N/A	Uncovered: uncovered nitinol metal stent (Nitinella, ELLA-CS, Hradec Kralove, Czech Republic) Covered: polycarbonate-polyurethane covered nitinol stent (Nitinella, ELLA-CS, Hradec Kralove, Czech Republic)
Telford	2010	USA	2002–2008	Uncovered: 61 Covered: 68	Uncovered: 65 ± 13 Covered: 66 ± 14	Uncovered: 50.8 Covered: 44.1	Karnofsky performance score Uncovered: mean 74 (SD, 17) Covered: mean 77 (SD, 18)	Uncovered: pancreatic cancer (77.0%), others (33.0%) Covered: pancreatic cancer (86.8%), others (13.2%)	N/A	Uncovered: uncovered Wallstent (Boston Scientific Corporation, Natick, MA, USA) Covered: permalume partially covered Wallstent (Boston Scientific Corporation, Natick, MA, USA)
Isayama	2011	Japan	2005–2007	Plastic: 58 Covered: 55	Plastic: 69.6 (range, 44–86) Covered: 71.1 (range, 53–86)	Plastic: 51.7 Covered: 60.0	N/A	Plastic: pancreatic cancer (100.0%) Covered: pancreatic cancer (100.0%)	N/A	Plastic: 10-Fr DLS duodenum bending type Tannenbaum (CWS, Microvesive, Boston Scientific, Natick, MA, USA) Covered: Covered Wallstent (CWS, Microvesive, Boston Scientific, Natick, MA, USA)
Krokidis	2011	Italy and Greece	2005–2008	Uncovered: 40 Covered: 40	Uncovered: median 65 (SD 8.8) Covered: median 63.5 (SD 9.8)	Uncovered: 90.0 Covered: 42.5	N/A	Uncovered: pancreatic cancer (100.0%) Covered: pancreatic cancer (100.0%)	Uncovered: median 8.3 (SD, 1.1) Covered: median 6.1 (SD, 1.3)	Uncovered: Luminexx Nitinol Biliary Stent (Bard, Murray Hill, NJ, USA) Covered: Viabil Biliary Stent (Goree, Flagstaff, AZ, USA)
Kitano	2013	Japan	2009–2010	Uncovered: 60 Covered: 60	Uncovered: 68.7 ± 8.9 Covered: 70.6 ± 10.7	Uncovered: 48.3 Covered: 41.7	N/A	Uncovered: pancreatic cancer (100.0%) Covered: pancreatic cancer (100.0%)	Uncovered: 5.3 ± 6.0 Covered: 5.1 ± 4.8	Uncovered: uncovered Wallflex biliary RX stent (Boston Scientific, Natick, MA, USA) Covered: covered Wallflex biliary RX stent (Boston Scientific, Natick, MA, USA)
Moses	2013	USA and Canada	N/A	Plastic: 41 Covered: 41	Plastic: 73.3 ± 10.7 Covered: 70.8 ± 12.9	Plastic: 50.0 Covered: 51.2	Karnofsky performance score Plastic: mean 82.0 (SD, 12.0) Covered: 81.8 (SD, 10.8)	Plastic: pancreatic cancer (67.5%), bile duct cancer (5.0%), gallbladder cancer (2.5%), ampullary cancer (7.5%), metastatic cancer (7.5%), others (2.5%) Covered: pancreatic cancer (69.2%), bile duct cancer (0.0%), gallbladder cancer (2.6%), ampullary cancer (2.6%), metastatic cancer (10.3%), others (0.0%)	Plastic: 11.3 ± 7.8 Covered: 9.6 ± 7.0	Plastic: 10-Fr Amsterdam-type polyethylene plastic stent Covered: partially covered Wallstent (Boston Scientific, Natick, MA, USA)
Ung	2013	Sweden	2006–2009	Uncovered: 34 Covered: 34	Uncovered: median 79 (IQR, 64–83) Covered: median 77 (IQR, 67–83)	Uncovered: 26.5 Covered: 52.9	N/A	Uncovered: pancreatic cancer (79.4%), gallbladder cancer (14.7%), ampullary cancer (8.8%), others (2.9%) Covered: pancreatic cancer: (88.2%), gallbladder cancer (5.9%), ampullary cancer (2.9%), others (5.9%)	Uncovered: median 10.6 (IQR, 1.7–14.2) Covered: median 4.6 (IQR, 1.8–15.2)	Uncovered: uncovered Hanarostent (MI-tech, Seoul, Korea) Covered: covered Hanarostent (MI-tech, Seoul, Korea)
Lee	2014	Korea	2012–2013	Uncovered: 20 Covered: 20	Uncovered: 63.2 ± 11.7 Covered: 62.1 ± 8.6	Uncovered: 45.0 Covered: 45.0	N/A	Uncovered: pancreatic cancer (30.0%), bile duct cancer (5.0%), gallbladder cancer (15.0%), others (50.0%) Covered: pancreatic cancer (60.0%), bile duct cancer (5.0%), gallbladder cancer (0.0%), others (35.0%)	Uncovered: 8.0 ± 6.6 Covered: 6.2 ± 4.9	Uncovered: Zilver self-expanding stent (Cook, Bloomington, IN, USA) Covered: Niti-S stent, ComVi type (Taewoong Medical Co., Ltd., Seoul, Korea)
Walter	2015	Netherlands	2008–2013	Plastic: 57 Uncovered: 60 Covered: 54	N/A	N/A	N/A	N/A	N/A	Plastic: 10-Fr polyurethane or polyethylene stent (Boston Scientific Corporation, Natick, MA, USA) Uncovered: 10-mm uncovered Wallstent RX (Boston Scientific Corporation, Natick, MA, USA) Covered: 10-mm covered Wallstent RX (Boston Scientific Corporation, Natick, MA, USA)
Yang	2015	Korea	2006–2013	Uncovered: 52 Covered: 51	Uncovered: 68.0 ± 11.3 Covered: 68.7 ± 11.2	Uncovered: 57.7 Covered: 66.7	Karnofsky performance score Uncovered: mean 79.4 (SD, 7.3) Covered: mean 78.0 (SD, 9.0)	Uncovered: pancreatic cancer (69.2%), bile duct cancer (13.5%), gallbladder cancer (9.6%), ampullary cancer (3.8%), others (3.8%) Covered: pancreatic cancer (56.9%), bile duct cancer (33.3%), gallbladder cancer (3.9%), ampullary cancer (3.9%), others (2.0%)	Uncovered: 11.4 ± 6.1 Covered: 10.7 ± 8.4	Uncovered: Uncovered Bonastent (Standard Sci-Tech Inc., Seoul, Korea) Covered: partially covered Bonastent (Standard Sci-Tech Inc., Seoul, Korea)
Bernon	2018	South Africa	2009–2013	Plastic: 19 Uncovered: 21	Plastic: median 65 (IQR, 60–80) Uncovered: median 69.5 (IQR, 59.5–74)	Plastic: 42.1 Uncovered: 42.9	ECOG performance status Plastic (0/1/2): 0.0%/21.1%/78.9% Uncovered (0/1/2): 4.8%/23.8%/71.4%	Plastic: pancreatic cancer (89.5%), bile duct cancer (10.5%) Uncovered: pancreatic cancer (85.7%), bile duct cancer (14.3%)	Plastic: median 19.8 (range, 4.2–38.1) Uncovered: median 20.9 (range, 2.3–39.8)	Plastic: standard polyethylene plastic stent (Boston Scientific, Natick, MA, USA) Uncovered: 10-mm uncovered stent (Boston Scientific, Natick, MA, USA)
Conio	2018	Italy	2014–2016	Uncovered: 80 Covered: 78	Uncovered: median 80 (range, 49–101) Covered: median 77.5 (range, 45–98)	Uncovered: 46.3 Covered: 50.0	N/A	Uncovered: pancreatic cancer (72.5%), bile duct cancer (17.5%), gallbladder cancer (1.3%), ampullary cancer (8.8%) Covered: pancreatic cancer (75.6%), bile duct cancer(10.3%), gallbladder cancer (6.4%), ampullary cancer (7.7%)	Uncovered: 11.1 ± 6.7 Covered: 10.5 ± 8.7	Uncovered: Niti-S D-type stent (Taewoong Medical Co. Ltd., Goyang, Korea) Covered: Niti-S biliary ComVi stent (Taewoong Medical Co. Ltd., Goyang, Korea)
Dhondt	2020	Belgium	2002–2018	Uncovered: 78 Covered: 73	Uncovered: 69 (range, 22–88) Covered: 68 (range, 35–90)	Uncovered: 43.6 Covered: 41.1	N/A	Uncovered: pancreatic cancer (57.7%), bile duct cancer (2.6%), duodenal cancer (1.3%), metastatic cancer (38.5%) Covered: pancreatic cancer (61.6%), bile duct cancer (0.0%), duodenal cancer (0.0%), metastatic cancer (38.4%)	Uncovered: 13.0 ± 0.9 Covered: 13.0 ± 0.8	Uncovered: ZA biliary stent (Cook Europe, Limerick, Ireland) Covered: VIABIL endoprosthesis (W.L. Gore & Associates, Flagstaff, Arizona, USA)

ECOG, Eastern Cooperative Oncology Group; ASA PS, American Society of Anesthesiologist physical status; SD, standard deviation; IQR, interquartile range; CI, confidence interval; N/A, not available.

## Data Availability

All relevant data are included in the study and supplementary information.

## Supplementary Materials

The following are available online at https://www.mdpi.com/2075-4426/11/2/86/s1, Figure S1: Evidence network of different stents. Lines represent the comparison between stent types. The thickness of these lines and the numbers shown in large represent the number of studies included in each comparison. The numbers in rectangles indicate the number of patients included in each comparison. Because one study was a three-arm trial, the sum of comparisons and that of patients exceeded the number of included studies and that of included patients, respectively. SEMS, self-expandable metal stent. Figure S2: Risk of bias summary: the review authors’ judgments regarding the risk of bias items for each included study. Figure S3: Funnel plot for the analysis of recurrent biliary obstruction between the uncovered and covered SEMS groups. Figure S4: Direct meta-analysis for causes of recurrent biliary obstruction. (A) sludge, (B) tumor ingrowth, (C) tumor overgrowth, and (D) stent migration. Figure S5: Direct meta-analysis for stent-related inflammation. (A) cholangitis, (B) cholecystitis, and (C) pancreatitis. Table S1: Network estimates of the palliative bile duct stent in terms of recurrent biliary obstruction and adverse events. Table S2: Incidence of recurrent biliary obstruction and adverse events according to the stent type.

## Appendix A. Detailed Search Strategy

MEDLINE (Pubmed)

(bile duct[Title/Abstract] OR biliary[Title/Abstract] OR cholangiocarcinoma[Title/Abstract] OR cholangiocarcinomas[Title/Abstract] OR pancreatic[Title/Abstract] OR pancreas[Title/Abstract]) AND (obstruction[Title/Abstract] OR obstructive[Title/Abstract] OR stricture[Title/Abstract] OR strictures[Title/Abstract] OR jaundice[Title/Abstract] OR cholestasis[Title/Abstract]) AND (nonresectable[Title/Abstract] OR unresectable[Title/Abstract] OR palliation[Title/Abstract] OR palliative[Title/Abstract] OR palliating[Title/Abstract] OR inoperable[Title/Abstract]) AND (stent[Title/Abstract] OR stents[Title/Abstract] OR stenting[Title/Abstract] OR endoprostheses[Title/Abstract]) AND (Randomized Controlled Trial[Publication Type] OR random*[Title/Abstract]) AND (“1990/01/01”[Date—Publication]: “2020/01/15”[Date—Publication])

EMBASE (Ovid)

1: ((‘bile duct’ or biliary or cholangiocarcinoma or cholangiocarcinomas or pancreatic or pancreas) and (obstruction or obstructive or stricture or strictures or jaundice or cholestasis) and (nonresectable or unresectable or palliation or palliative or palliating or inoperable) and (stent or stents or stenting or endoprostheses) and random*).ab, ti.

2: Limit 1 to (yr = “1990 -Current”)

Cochrane library

#1: ‘bile duct’ or biliary or cholangiocarcinoma or cholangiocarcinomas or pancreatic or pancreas

#2: obstruction or obstructive or stricture or strictures or jaundice or cholestasis

#3: nonresectable or unresectable or palliation or palliative or palliating or inoperable

#4: stent or stents or stenting or endoprostheses

#5: random*

#6: #1 and #2 and #3 and #4 and #5 (with Publication Year from 1990 to 2020, in Trials)
