# Supplementary material for: Comparative Efficacy of Various Stents for Palliation in Patients with Malignant Extrahepatic Biliary Obstruction: A Systematic Review and Network Meta-Analysis
Source: J Pers Med. 2021 Jan 30;11(2):86. doi: 10.3390/jpm11020086 (PMC7912345; doi:10.3390/jpm11020086)
Supplement: Supplementary file 1 [file jpm-11-00086-s001.zip › Table S1.docx]

| Table S1. Network estimates of the palliative bile duct stent in terms of recurrent biliary obstruction and adverse events. | | | | |
| --- | --- | --- | --- | --- |
| Outcome | Stent type | Compared stent type | | |
|  |  | vs. plastic stent RR (95% CI) | vs. uncovered SEMS RR (95% CI) | vs. covered SEMS RR (95% CI) |
| RBO |  |  |  |  |
|  | Plastic stent | . | 2.15 (1.62–2.87) | 2.19 (1.62–2.97) |
|  | Uncovered SEMS | 0.46 (0.35–0.62) | . | 1.02 (0.80–1.30) |
|  | Covered SEMS | 0.46 (0.34–0.62) | 0.98 (0.77–1.25) | . |
| Occlusion by sludge |  |  |  |  |
|  | Plastic stent | . | 11.70 (5.43–25.23) | 5.94 (2.69–13.13) |
|  | Uncovered SEMS | 0.09 (0.04–0.18) | . | 0.51 (0.31–0.83) |
|  | Covered SEMS | 0.17 (0.08–0.37) | 1.97 (1.20–3.23) | . |
| Tumor ingrowth |  |  |  |  |
|  | Plastic stent | . | 0.04 (0.00–0.39) | 0.19 (0.02–1.78) |
|  | Uncovered SEMS | 23.79 (2.54–222.81) | . | 4.49 (2.21–9.09) |
|  | Covered SEMS | 5.30 (0.56–50.01) | 0.22 (0.11–0.45) | . |
| Tumor overgrowth |  |  |  |  |
|  | Plastic stent | . | 0.32 (0.04–2.83) | 0.16 (0.02–1.34) |
|  | Uncovered SEMS | 3.17 (0.35–28.42) | . | 0.50 (0.30–0.85) |
|  | Covered SEMS | 6.29 (0.75–52.89) | 1.98 (1.18–3.34) | . |
| Stent migration |  |  |  |  |
|  | Plastic stent | . | 3.45 (0.86–13.84) | 0.41 (0.13–1.34) |
|  | Uncovered SEMS | 0.29 (0.07–1.16) | . | 0.12 (0.04–0.35) |
|  | Covered SEMS | 2.43 (0.74–7.94) | 8.38 (2.83–24.84) | . |
| Cholangitis |  |  |  |  |
|  | Plastic stent | . | 1.59 (0.89–2.84) | 2.37 (1.003–5.60) |
|  | Uncovered SEMS | 0.63 (0.35–1.13) | . | 1.49 (0.73–3.07) |
|  | Covered SEMS | 0.42 (0.18–0.997) | 0.67 (0.33–1.38) | . |
| Cholecystitis |  |  |  |  |
|  | Plastic stent | . | 0.69 (0.14–3.31) | 0.43 (0.10–1.91) |
|  | Uncovered SEMS | 1.46 (0.30–7.04) | . | 0.63 (0.31–1.28) |
|  | Covered SEMS | 2.30 (0.52–10.12) | 1.58 (0.78–3.19) | . |
| Pancreatitis |  |  |  |  |
|  | Plastic stent | . | 2.64 (0.62–11.35) | 1.42 (0.35–5.77) |
|  | Uncovered SEMS | 0.38 (0.09–1.63) | . | 0.54 (0.22–1.33) |
|  | Covered SEMS | 0.70 (0.17–2.84) | 1.86 (0.75–4.58) | . |
| RBO, recurrent biliary obstruction; SEMS, self-expandable metal stent; RR, risk ratio; CI, confidence interval. | | | | |
