# Supplementary material for: Comparative Efficacy of Various Stents for Palliation in Patients with Malignant Extrahepatic Biliary Obstruction: A Systematic Review and Network Meta-Analysis
Source: J Pers Med. 2021 Jan 30;11(2):86. doi: 10.3390/jpm11020086 (PMC7912345; doi:10.3390/jpm11020086)
Supplement: Supplementary file 1 [file jpm-11-00086-s001.zip › Table S2.docx]

| Table S2. Incidence of recurrent biliary obstruction and adverse events according to the stent type. | | | |
| --- | --- | --- | --- |
| Outcome | Crude proportion (95% CI) | | |
|  | Plastic stent | Uncovered SEMS | Covered SEMS |
| RBO | 47.4% (42.8%–52.1%) | 23.8% (21.3%–26.7%) | 23.6% (20.9%–26.6%) |
| Occlusion by sludge | 44.8% (37.3%–-52.5%) | 3.4% (2.3%–5.1%) | 7.5% (5.8%–9.8%) |
| Tumor ingrowth | 0.0% (0.0%–6.6%) | 17.3% (14.5%–20.4%) | 3.1% (2.0%–4.7%) |
| Tumor overgrowth | 0.0% (0.0%–0.7%) | 3.4% (2.2%–5.2%) | 7.1% (5.4%–9.3%) |
| Stent migration | 3.1% (1.4%–6.7%) | 0.0% (0.0%–1.2%) | 5.0% (3.6%–6.9%) |
| Cholangitis | 36.3% (29.4%–43.7%) | 12.1% (9.5%–15.4%) | 5.2% (3.4%–7.9%) |
| Cholecystitis | 0.5% (0.1%–3.6%) | 1.6% (0.9%–2.8%) | 3.1% (2.1%–4.6%) |
| Pancreatitis | 2.3% (1.0%–5.5%) | 1.0% (0.5%–2.3%) | 2.3% (1.4%–3.8%) |
| RBO, recurrent biliary obstruction; SEMS, self-expandable metal stent; CI, confidence interval. | | | |
