# Supplementary figures and images for: Comparative Efficacy of Various Stents for Palliation in Patients with Malignant Extrahepatic Biliary Obstruction: A Systematic Review and Network Meta-Analysis
Source: J Pers Med. 2021 Jan 30;11(2):86. doi: 10.3390/jpm11020086 (PMC7912345; doi:10.3390/jpm11020086)

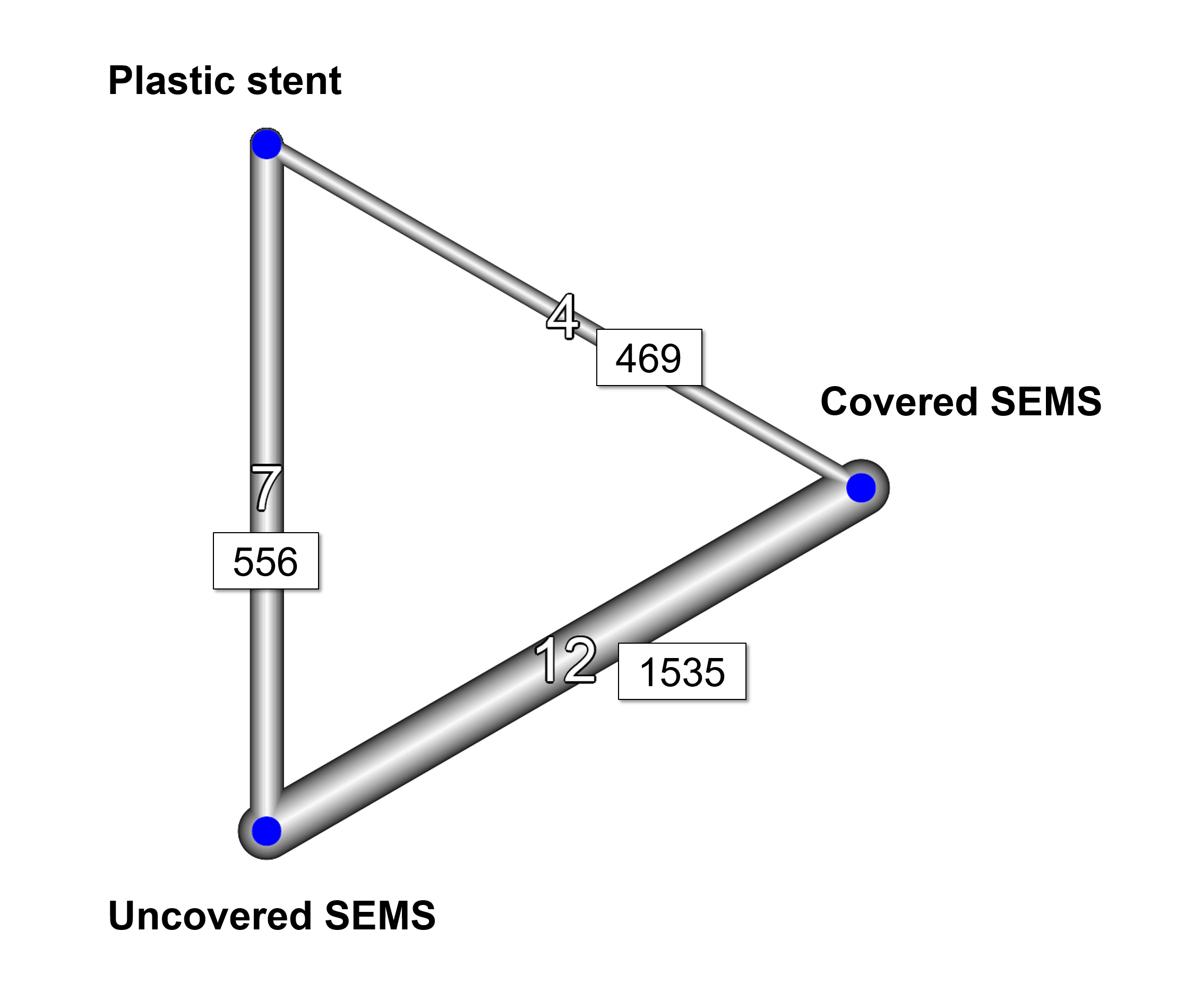

Supplement: Supplementary file 1 [file jpm-11-00086-s001.zip › Figure S1.tif]

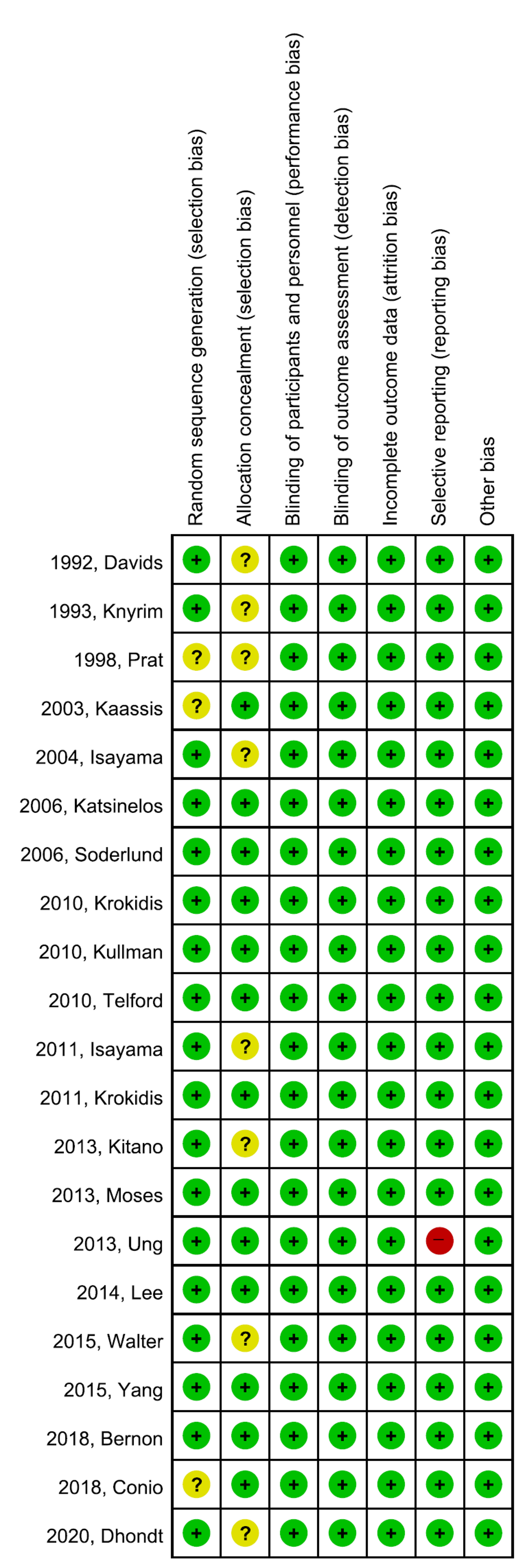

Supplement: Supplementary file 1 [file jpm-11-00086-s001.zip › Figure S2.tif]

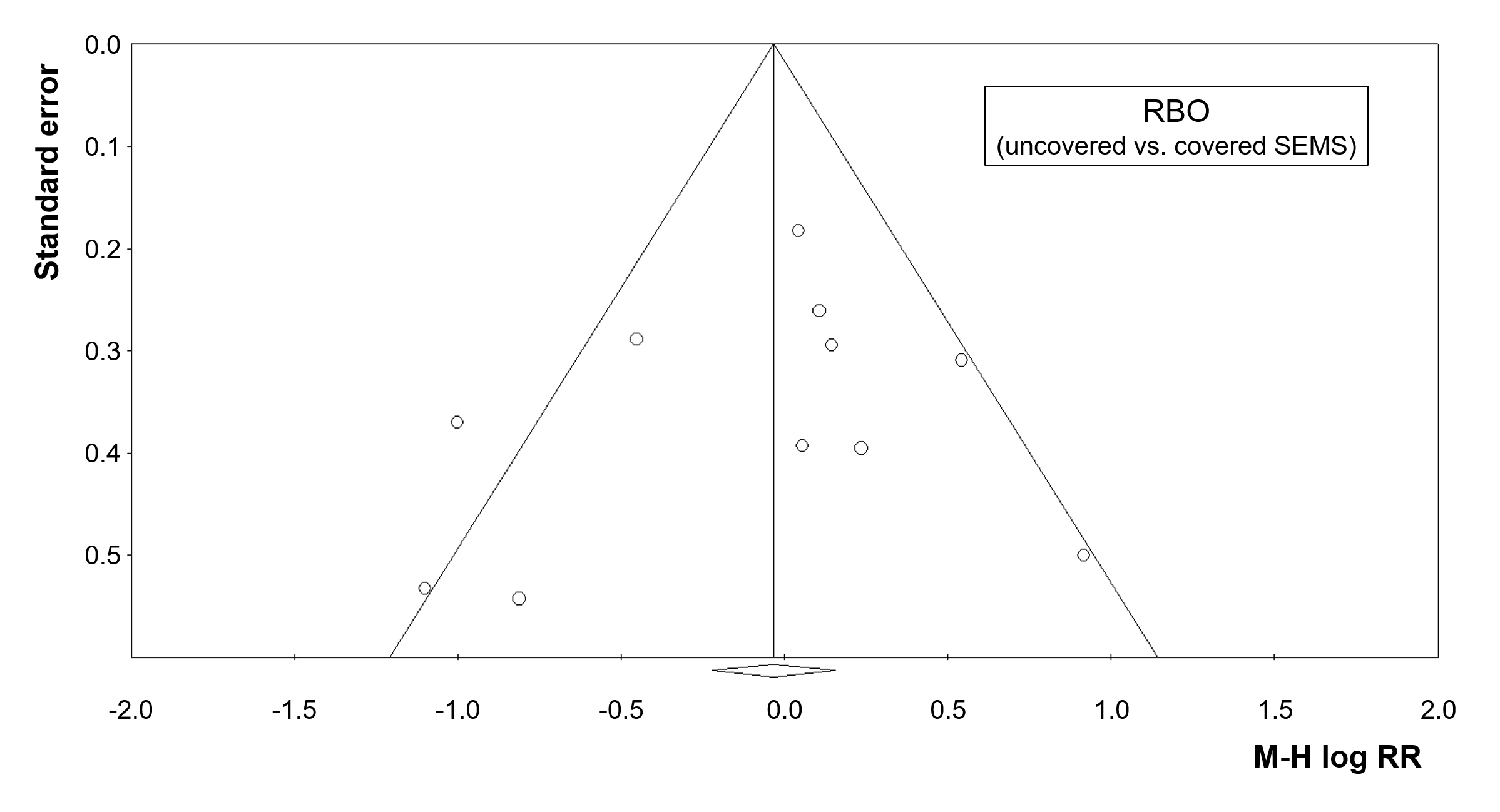

Supplement: Supplementary file 1 [file jpm-11-00086-s001.zip › Figure S3.tif]

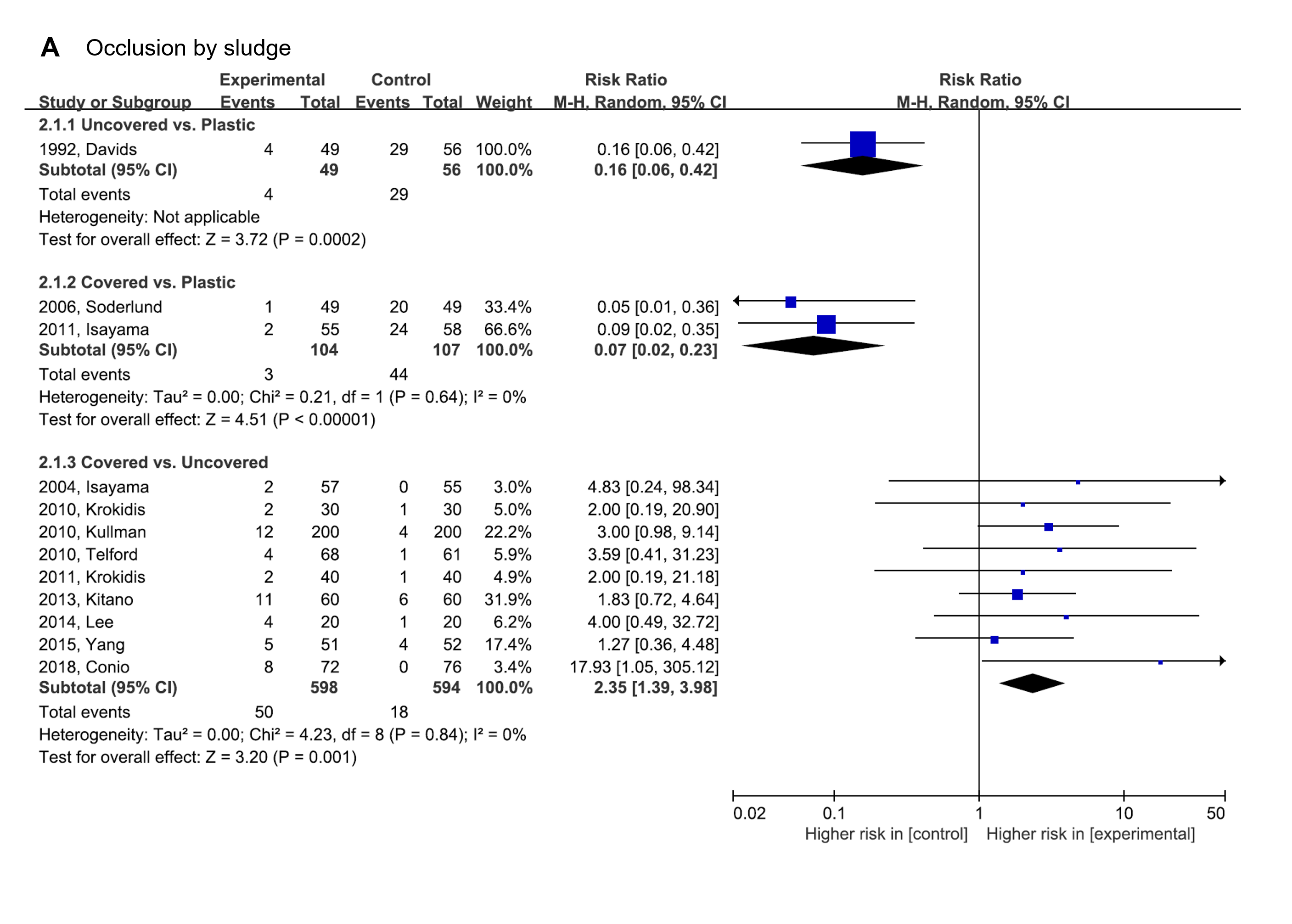

Supplement: Supplementary file 1 [file jpm-11-00086-s001.zip › Figure S4A.tif]

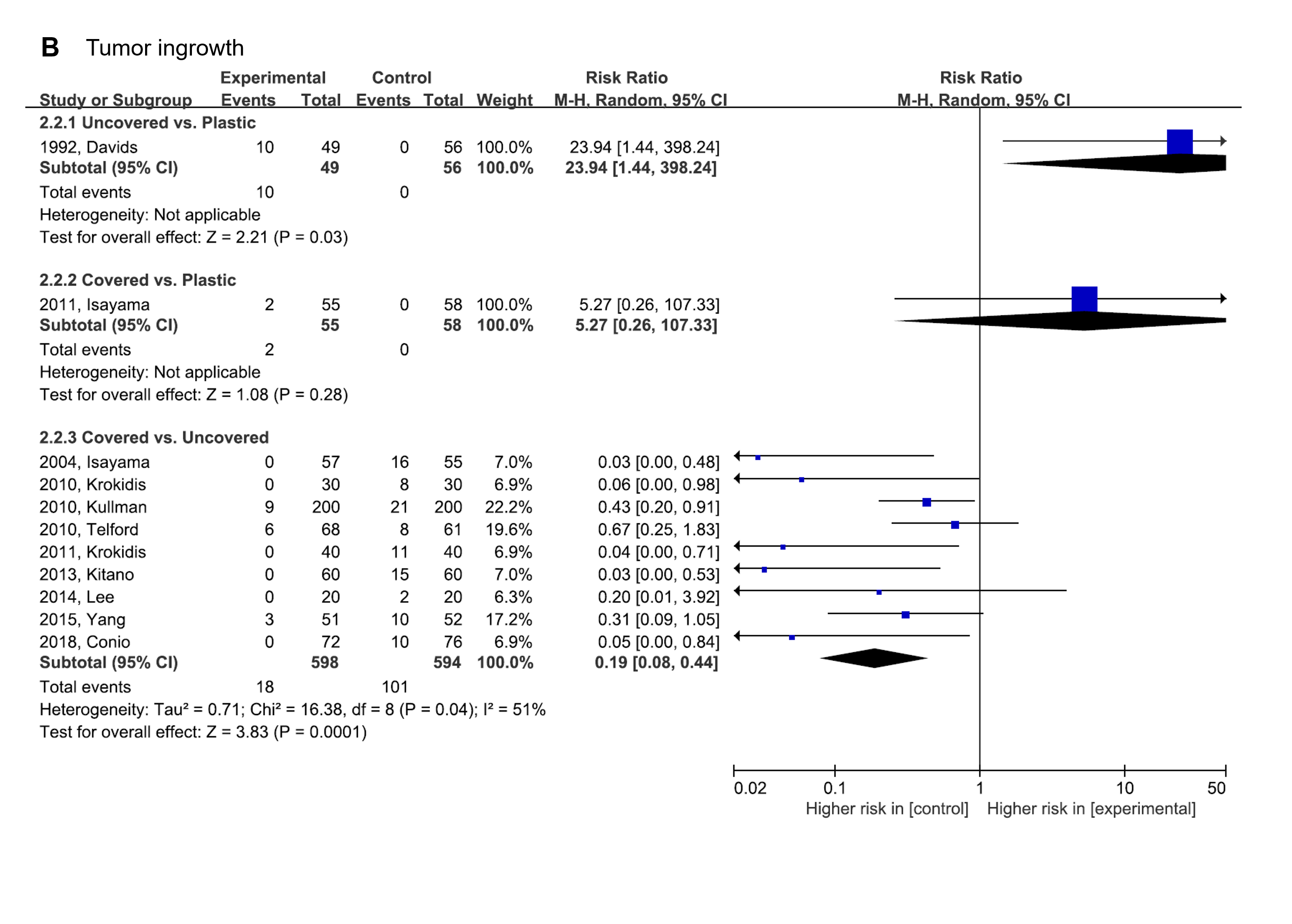

Supplement: Supplementary file 1 [file jpm-11-00086-s001.zip › Figure S4B.tif]

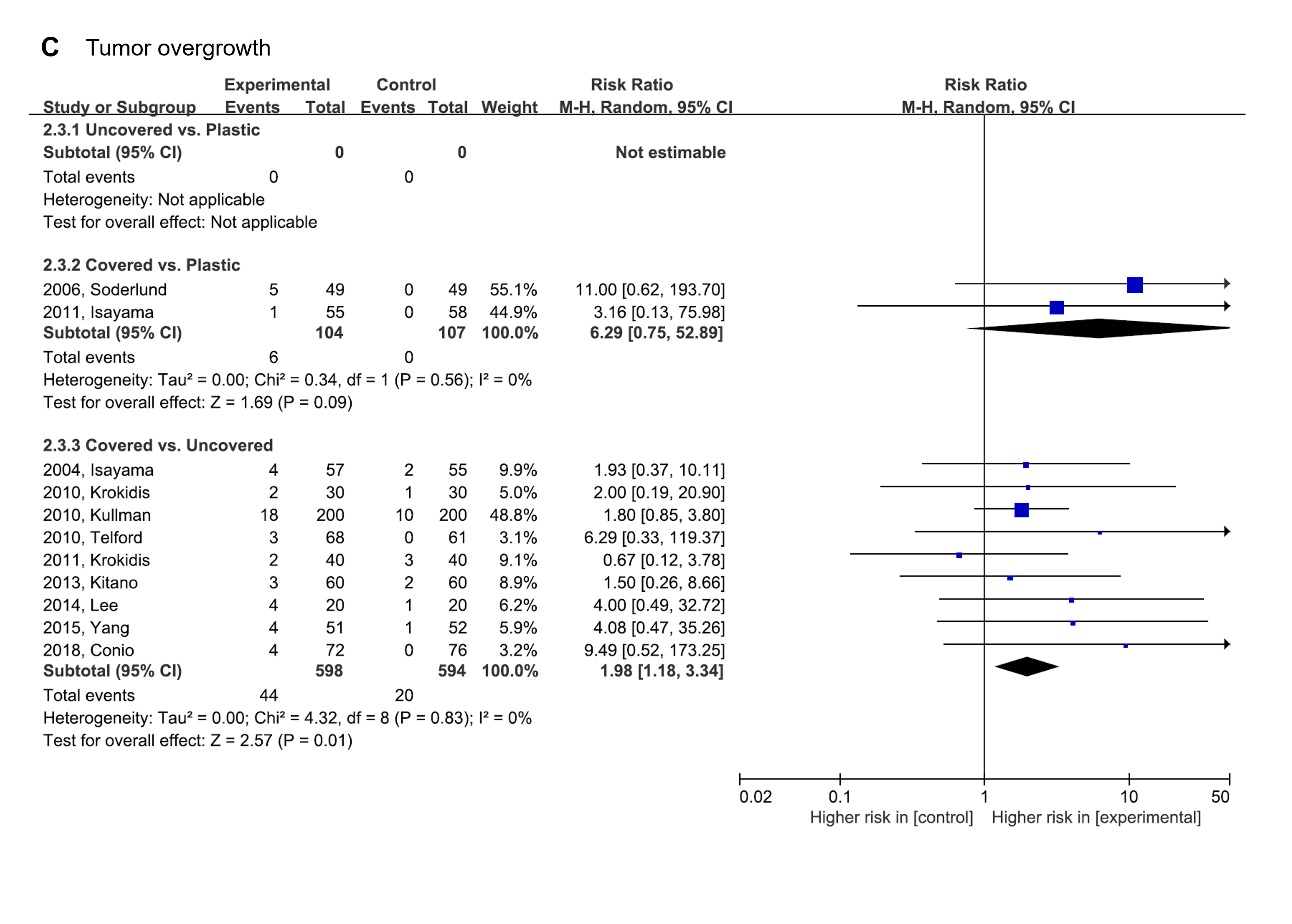

Supplement: Supplementary file 1 [file jpm-11-00086-s001.zip › Figure S4C.tif]

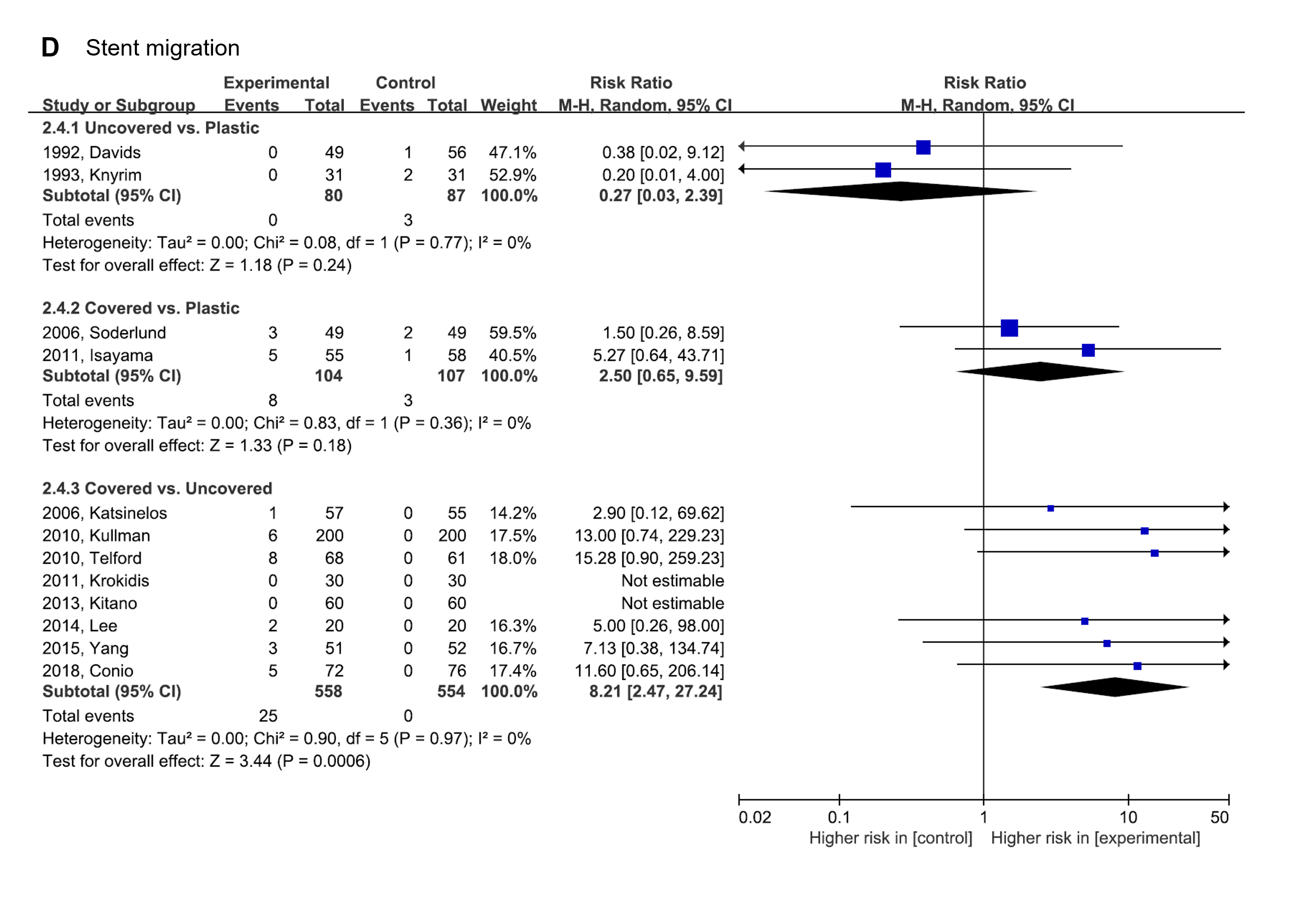

Supplement: Supplementary file 1 [file jpm-11-00086-s001.zip › Figure S4D.tif]

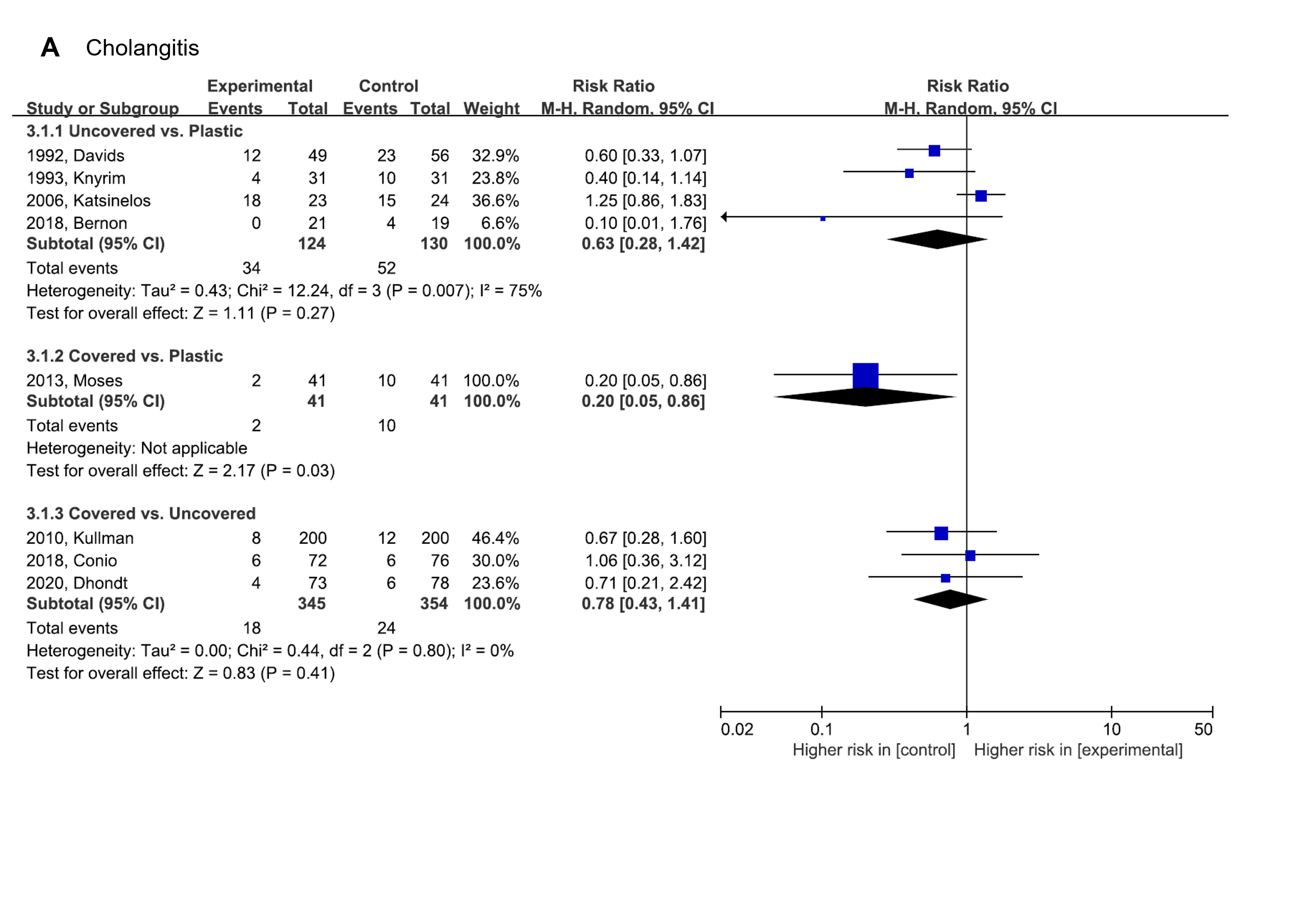

Supplement: Supplementary file 1 [file jpm-11-00086-s001.zip › Figure S5A.tif]

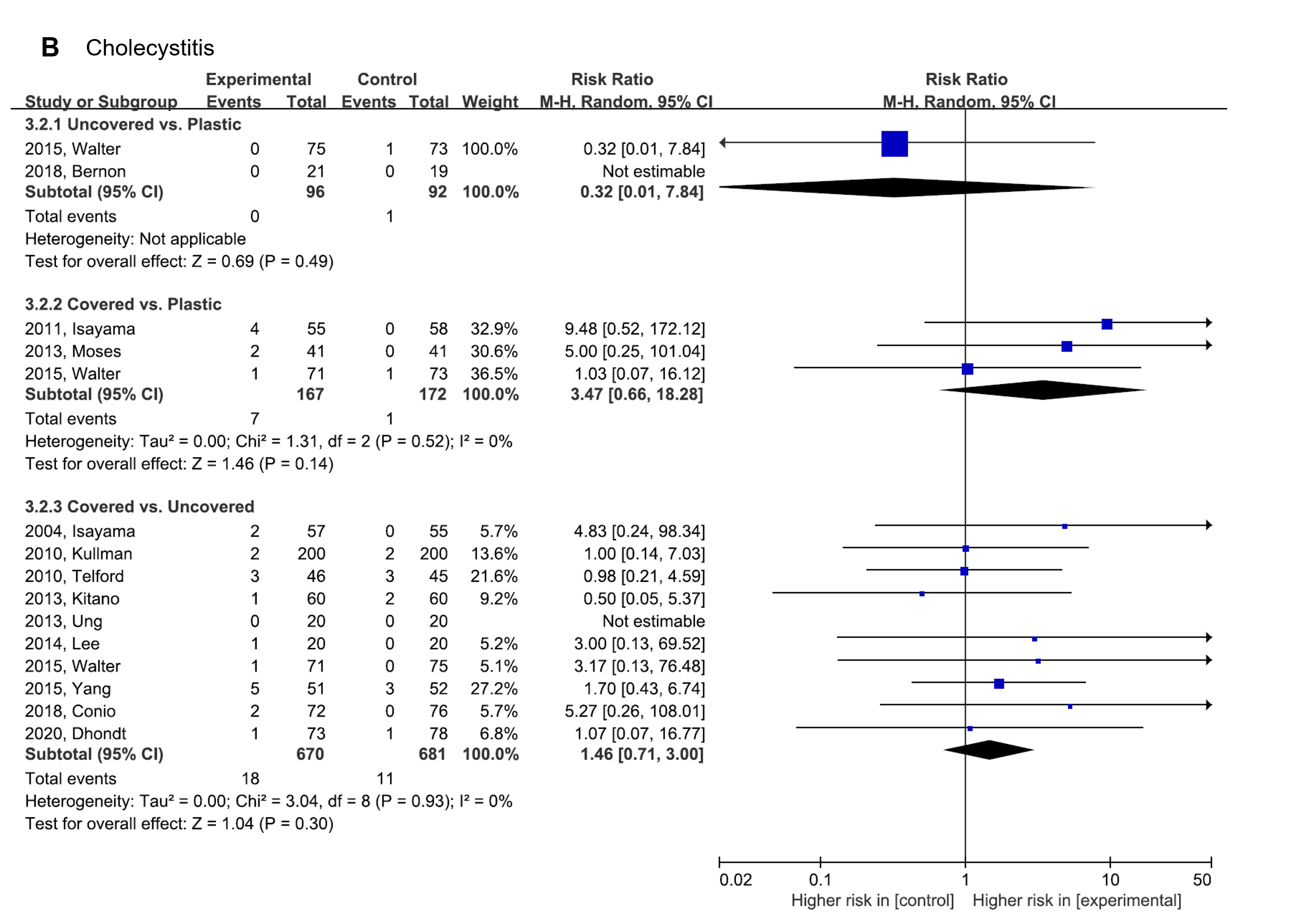

Supplement: Supplementary file 1 [file jpm-11-00086-s001.zip › Figure S5B.tif]

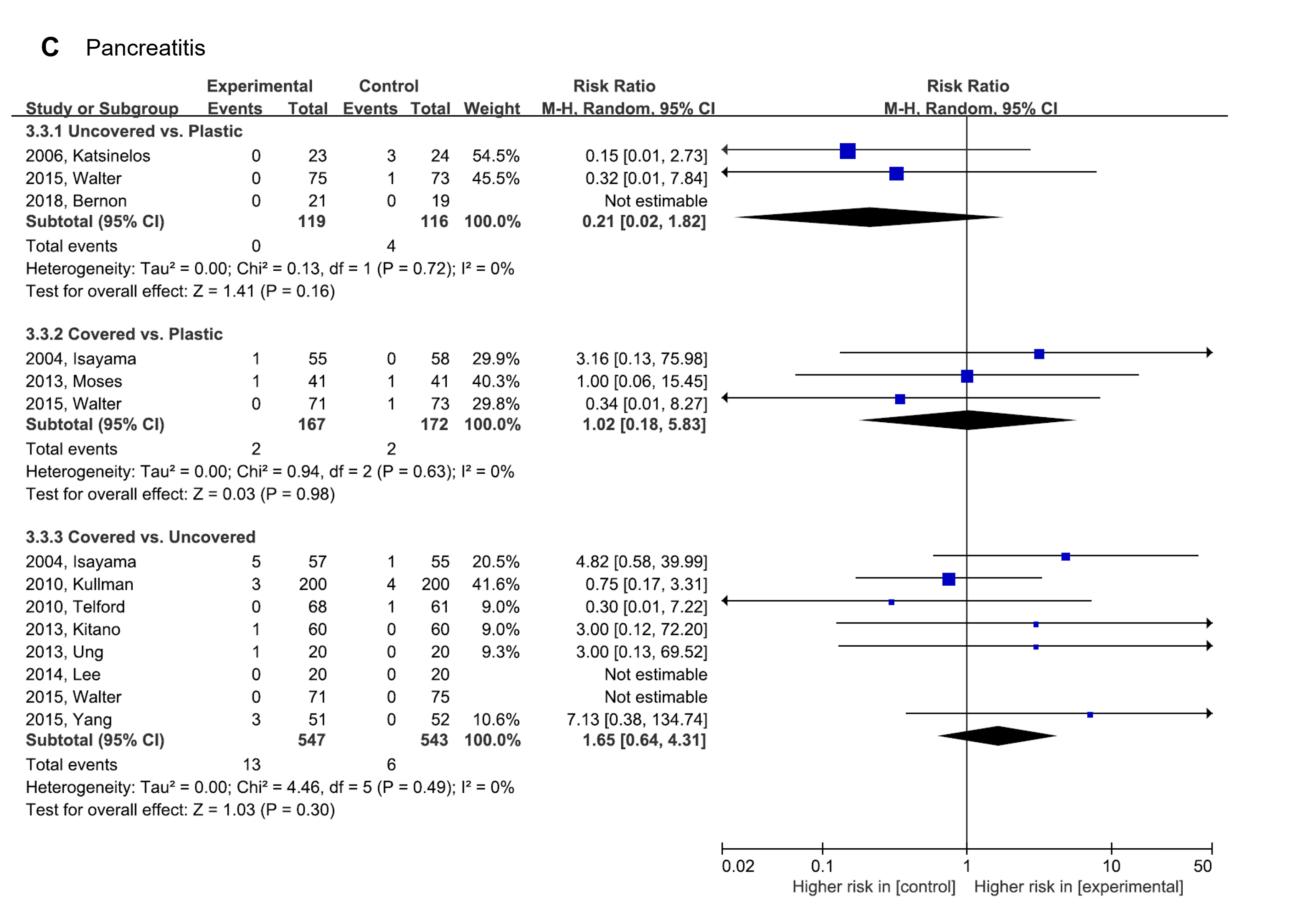

Supplement: Supplementary file 1 [file jpm-11-00086-s001.zip › Figure S5C.tif]
